# The genetics of Graves’ disease

**DOI:** 10.1007/s11154-023-09848-8

**Published:** 2023-12-18

**Authors:** Lydia Grixti, Laura C. Lane, Simon H Pearce

**Affiliations:** 1 Translational and Clinical Research Institute, Newcastle University, BioMedicine West, Central Parkway, Newcastle-upon-Tyne, NE1 3BZ UK; 2 Endocrine Unit, Royal Victoria Infirmary, Queen Victoria Road, Newcastle-upon-Tyne, NE1 4LP UK; 3 Department of Paediatric Endocrinology, The Great North Children’s Hospital, Queen Victoria Road, Newcastle-upon-Tyne, NE1 4LP UK

**Keywords:** Immunogenetics, Autoimmune thyroid disease, Genome-wide association, Graves’ disease, Thyroid eye disease, Genotype-phenotype correlation

## Abstract

Graves’ disease (GD) is the commonest cause of hyperthyroidism and has a strong female preponderance. Everyday clinical practice suggests strong aggregation within families and twin studies demonstrate that genetic factors account for 60-80% of risk of developing GD. In this review, we collate numerous genetic studies and outline the discoveries over the years, starting with historic candidate gene studies and then exploring more recent genome-wide linkage and association studies, which have involved substantial cohorts of East Asian patients as well as those of European descent. Variants in genes including *HLA*, *CTLA4*, and *PTPN22* have been shown to have substantial individual effects on disease susceptibility. In addition, we examine emerging evidence concerning the possibility that genetic variants may correlate with relevant clinical phenotypes including age of onset of GD, severity of thyrotoxicosis, goitre size and relapse of hyperthyroidism following antithyroid drug therapy, as well as thyroid eye disease. This review supports the inheritance of GD as a complex genetic trait, with a growing number of more than 80 susceptibility loci identified so far. Future implementation of more targeted clinical therapies requires larger studies investigating the influence of these genetic variants on the various phenotypes and different outcomes of conventional treatments.

## Epidemiology and heritability

Graves' disease (GD) is the commonest cause of hyperthyroidism affecting approximately 3% of women and 0.5% of men during their lifetime [1]. GD, in common with most other autoimmune diseases, has a clear female preponderance with a female to male ratio of 6-7:1. The incidence was 0.04% per year during a 12-year follow-up of the Nurses Health II study in North America [2]. Interestingly, a recent longitudinal study of 22 million people from the UK Clinical Practice Research Datalink showed a doubling in incidence of GD, from 0.03% per year in 2000-2002 to 0.07% per year by 2017-2019 [3]. In members of the US armed forces, Graves’ disease occurred nearly twice as commonly in Black and Asian/Pacific Islander women than in White women [4].

Twin studies in the Danish and Swedish populations suggest strong genetic aggregation, with a concordance rate for monozygotic twins of between 20% and 35%, compared to 2-3% in dizygotic twins [5, 6], fitting a model where genetic factors account for 60-80% of the risk of developing GD [5, 6]. An estimate of heritability can also be given by the sibling recurrence risk ratio, known as λs. A Hungarian study showed that 23 of 435 (5.3%) GD probands had siblings with GD (21 sisters, 2 brothers), compared with a background population frequency of GD of 0.65% [7]. A similar study performed in Newcastle, UK showed 15 of 190 (7.9%) GD probands had similarly affected siblings (9 sisters, 6 brothers), compared with a background prevalence of GD of 0.8% [8]. This allows the calculation of λs for GD as 8–10, which compares to that of 8 for rheumatoid arthritis, 15 for type 1 diabetes and 20 for multiple sclerosis [9]. This supports that GD, in common with many other autoimmune conditions is inherited as a complex genetic trait, with strong familial clustering but without a classical Mendelian pattern of inheritance [5, 6, 8, 9].

A key question given the large 60-80% genetic contribution to disease susceptibility is what constitutes the remaining non-genetic or potential ‘precipitating’ factors. Although outside the scope of this review, a longitudinal study of Dutch people who had a sibling with autoimmune thyroid disease (AITD) showed that pregnancy was a risk factor for development of GD, whereas oestrogen use was protective [10]. In addition, there are high quality studies indicating that stress, including post-traumatic stress disorder, cigarette use and iodine status also contribute as non-genetic factors that determine the manifestation of GD [11–13].

Although there are a few studies that have investigated the evolving field of epigenetics of GD, several of these are small with inadequate power, and no independently replicated results have been found thus far [14–17]. For these reasons, the remainder of this review will focus on germline inherited genetic variation in Graves’ disease.

## Historical genetic studies: candidate gene studies and the trinity of organ specific autoimmunity

Early genetic studies focussed on alleles of the major histocompatibility complex (MHC), primarily because this was one of the first loci for which reliable markers could be used to infer genotype. Using serological assays for human leukocyte antigen (HLA) typing initially allowed strong association to be established between the MHC alleles (“HL-A8”) on the short arm of chromosome 6 and GD [18]. This has now been refined such that in populations of European decent, an associated haplotype HLA-DRB1*0301-DQB1*0201-DQA1*0501 has been confirmed in many different studies, with an odds ratio between 2 and 3 compared to the unaffected population [19, 20]. In Asian populations, different HLA associations have been confirmed, including DRB1*0405 and DRB1*1403 in Japanese [21, 22], DRB1*0803 and DRB1*1602 in Koreans [23], and DRB1*1602 in the Thai population [24]. The associated DRB1*0301 allele encodes for an arginine residue at position 74 of the MHC-DRβ chain, and this has been hypothesized to change the binding pocket of the MHC to facilitate binding of T lymphocyte peptide antigen [25]. Nevertheless, as only around 50% of GD patients of European ancestry carry the DRB1*0301 haplotype, it suggests that MHC-restricted presentation of a single antigenic peptide is unlikely to account for all cases of GD.

Following on from the identification of MHC as a GD locus, a candidate gene study examined a microsatellite repeat polymorphism in the then recently discovered cytotoxic T-lymphocyte antigen-4 (*CTLA4*) gene. *CTLA4* was an excellent candidate gene for an autoimmune disorder as a negative costimulatory molecule, present as a ‘second signal’ modulating T cell receptor activation in response to MHC-antigen presentation at the immunological synapse. Even though this study was relatively small with only 133 Graves’ patients [26], it was strongly positive and has been replicated on more than 50 occasions since then [26, 27]. In addition, association with *CTLA4* alleles has been confirmed in Type 1 diabetes, coeliac disease and numerous different organ-specific autoimmune conditions. Interestingly, the susceptibility allele for autoimmunity at CTLA4 occurs in more than 50% of the healthy population, and appears to be enriched in patients with thyroid eye disease [28].

Subsequent to its identification as a susceptibility allele for type 1 diabetes [29], a missense polymorphism (R620W) in the lymphoid tyrosine phosphatase (LYP, encoded by the *PTPN22* gene) was found to be associated with GD [30, 31]. This functions as another negative regulator of T lymphocyte signalling, and the associated allelic variant prevents the LYP protein from interacting with its inhibitory partner Csk, leading to unchecked TCR signalling. The PTPN22*620W allele is found in increasing frequency in Northern European countries as compared to populations in Southern Europe, and is virtually absent in Asian or African populations [23], suggesting that the variant allele may have been selected for ability to combat certain infectious disease.

Thus, by the end of the last century, alleles at the *MHC*, *CTLA4* and *PTPN22* loci had been robustly confirmed as contributing to GD susceptibility largely through the candidate gene approach. These same alleles had also been associated with Hashimoto’s thyroiditis and many different autoimmune disorders in which they were presumed to have an effect on immune system function. However, they did not explain why the thyroid gland is targeted by the immune response to give GD.

## Discovery-based genomics and early genome-wide association studies in European-ancestry populations

Although candidate gene studies were successful in identifying several GD susceptibility genes with large effects, the approach is limited in that one cannot discover an unsuspected biological basis for the disease using this approach. With this substantial limitation in mind, discovery-based approaches using genome-wide linkage analyses of families with two or more affected relatives with AITD were published by US and European investigators [32, 33]. The US study of 56 multigenerational families replicated the known linkage at *MHC* [34], and showed novel evidence for linkage at three other loci labelled as GD-1 (D14S81 on chromosome 14q31), GD-2 (D20S195 on chromosome 20q11.2) and GD-3 (DXS8020 on chromosome Xq21) [32]. The linkage to 14q31 and 20q11 was later replicated in a larger dataset of 102 multiplex families, although that to the X chromosome was not [35].

A disadvantage of family-based linkage studies over unrelated proband association analysis is that linkage identifies large chromosomal regions containing many potentially relevant genes and numerous allelic variants. The large gene region on 14q31 encompassing the GD-1 locus included the gene encoding the TSH receptor (TSHR), although consistent association was difficult to demonstrate in further studies. Subsequently, with an apparently un-ironic affiliation from “Target Discovery at Oxagen”, TSHR was confirmed to be a disease specific locus for GD using two independent European patient cohorts [36]. Non-coding variants within intron 1 of the TSHR gene were identified as being associated with GD by several groups [37, 38]. TSHR is expressed at low levels in the thymus presumably to allow T lymphocyte negative selection against TSHR as a “self antigen”, and people carrying the non-coding disease-associated variants were found to have lower levels of thymic TSHR mRNA expression. The hypothesis suggests that thymic T cells carrying T cell receptors specific for TSHR peptide may escape clonal deletion because of insufficient TSHR expression, leading to TSHR autoreactivity and thence GD [39].

Additional genome wide linkage studies were performed by the Oxagen investigators in 1,119 relative pairs of European origin with AITD [33] but failed to find significant linkage of any of the previously known loci including *MHC*, *CTLA4* and *PTPN22*. This study instead reported linkage with 3 regions for GD that included chromosomes 2q36, 11p15 and 18p11. The strongest region of linkage for GD was identified to marker D18S53 on chromosome 18p11 that had not been previously identified [33], and in retrospect this signal probably arose from the *PTPN2* gene, which has subsequently been confirmed as a GD locus. Similarly, a genome wide association scan published by the Wellcome Trust Case Control Consortium (WTCCC) included 1000 independent cases of GD, along with patients with ankylosing spondylitis, multiple sclerosis and breast cancer and compared these to 1500 randomly selected healthy British individuals from the 1958 Birth Cohort [40]. Associations were observed with SNPs in the MHC region with robust p-values of <10^-20^. Association was also reported and confirmed in GD patients at the *TSHR* locus and at a novel locus *FCRL3* [40]. The *FCRL3* association was rapidly replicated, not only in patients with GD [41], but also in rheumatoid arthritis and several other autoimmune conditions [41, 42]. Although the WTCCC study identified several other associations, at the time these could not be replicated [43]. That these two studies failed to replicate several previously confirmed loci was likely due to mixing cases of GD and Hashimoto thyroiditis together as well as low power, emphasizing that numerous loci contribute to complex autoimmune disease pathogenesis and that larger and more phenotypically homogeneous cohorts are needed to provide robust results.

## Expanding the genomic findings to Asian populations

In parallel with discovery-based genomic approaches in patients of European origin, investigators working in Japan and China began performing similar studies. Independent studies from both Japanese and Chinese groups using 123 and 54 multiplex families, respectively, both pointed to a susceptibility locus at chromosome 5q31-33 [44, 45]. Although initially suspected to relate to the presence of a cluster of interleukin genes (IL3, IL4, IL5 and GM-CSF) at this location, a more substantial association study in 2,800 Chinese Graves’ patients suggested that variants in the promoter of the Secretoglobin Family 3A Member 2 (SCGB3A2, encoding uterus globulin associated protein-1:UGRP1) gene might be responsible [46]. UGRP1 is secreted in lung surfactant and bronchial epithelia, is aberrantly expressed in thyroid tissue from both Hashimoto and GD patients, as well as being a downstream target of thyroid transcription factor-1 [47]. This finding of association with alleles of SCGB3A2 was quickly replicated in a UK cohort by Simmonds et al. indicating that this is also a susceptibility locus in Caucasian patients [48].

In a staged fashion over several years, the China Consortium for the Genetics of Autoimmune Thyroid Disease published larger association studies, culminating in a genome-wide analysis containing more than 9,500 individuals with GD [49, 50]. Cumulatively, these studies robustly identified eight new susceptibility alleles including at *RNASET2* (6q27), *GPR174* (Xq21.1), *C1QTNF6-RAC2* (22q12.3-13.1), *SLAMF6* (1q23.2), *ABO* (9q34.2), *Thyroglobulin* (8q24.22) and intergenic regions at 4p14 and 14q32.2 [49, 50]. In addition, associations at *MHC, CTLA4, FCRL3* and *TSHR* were confirmed [51]. The susceptibility variant in the *PTPN22* gene (620*W) is not present in Asian populations so association could not be directly replicated, however, there was modest evidence for a different allele at *PTPN22* having a role in Chinese Graves’ patients [52].

Another large three-stage genome wide association study carried out by Liu et al. in 2013 [52] showed that alleles of the *BACH2* gene were associated with GD in the Chinese Han population. *BACH2* had previously been identified as harbouring susceptibility variants in both Graves’ and Hashimoto’s thyroiditis patients in a smaller UK population [53]. A detailed analysis of 331-kb region in the *BACH2* gene in more than 3000 patients with GD and 1468 controls showed that a SNP (rs2474619) in intron 2 was most likely to be driving the disease association, and this variant was correlated with BACH2 gene expression. BACH2 deficient mice have defective immunoglobulin class switch recombination and BACH2 is a repressor of plasma cell differentiation, being essential for normal B cell maturation [54]. Additional studies in other autoimmune disorders have confirmed association of the same BACH2 alleles with Addison’s disease, type 1 diabetes, coeliac disease, and rheumatoid arthritis [55–57]. Thus, the large patient datasets particularly available to Chinese researchers allowed the robust identification of many novel GD loci during 2000-2010s which have subsequently been confirmed, as well as replication of some of the earlier associations found using the candidate gene approach.

## Large GWAS in European-ancestry populations

**Fig. 1:**
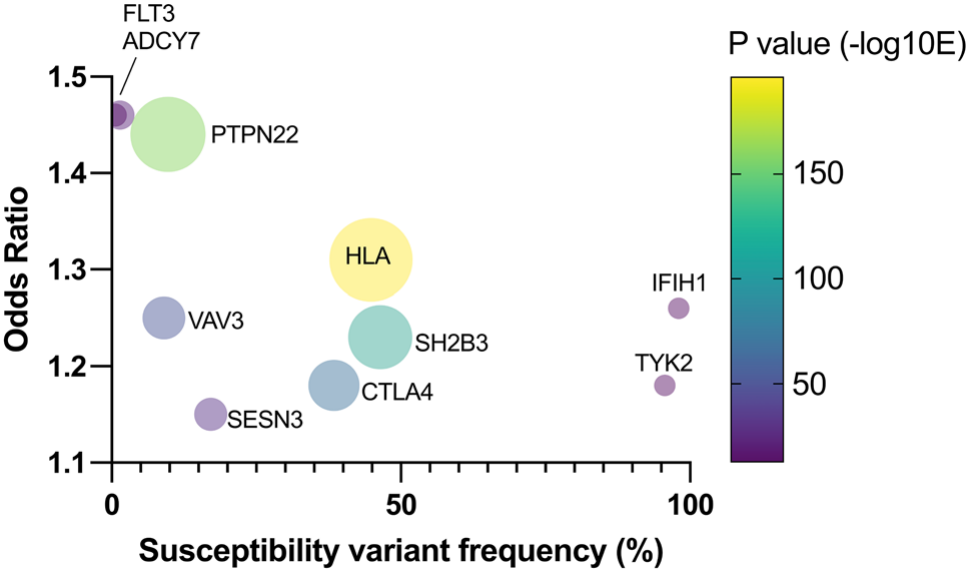
Genome-wide association scan in 30,000 patients with autoimmune thyroid disease- top 10 susceptibility loci. Data taken from Saevarsdottir et al. [58], show the balance between the odds ratio of the susceptibility allele predisposing to disease vs the allele frequency in the population. Bubble size/colour represents the probability in favour of association (-log10 P value). It is notable that probability for association is less for alleles with very low (FLT3, ADCY7) and very high (IFIH1, TYK2) population frequency, owing to low power.

A large genome-wide association study in a mixed cohort of 30,234 cases of AITD (both Graves’ and Hashimoto thyroiditis) and 72,172 controls from Iceland and the UK Biobank found 99 genome wide significant associations at 93 loci (Fig. 1)[58]. Of the 99 associations, 84 had not been previously associated with AITD. 37 candidate genes were supported by systematic annotation. 24 of these encoded proteins were found to be directly or indirectly linked in the same functional network. Interestingly, the study found two novel, but low frequency variants that showed comparatively large effects (Odds ratio around 1.5) on the susceptibility to AITD: an intronic variant in the *FLT3* gene (rs76428106-C) and a missense variant in *ADCY7* (rs78534766-A). *FLT3* gene variants had not previously been associated with any disease, whilst an *ADCY7* variant was previously reported to be associated with ulcerative colitis. The *FLT3* variant is intronic but introduces cryptic splice site and a premature stop codon predicting a truncated protein. The association was replicated in patient cohorts with systemic lupus erythematous, rheumatoid arthritis and coeliac disease [58].

Overall, only 15 of 99 variants had effect sizes with odds ratios over 1.10, and these comprised a mixture of common alleles (eg. *HLA, CTLA4, PTPN22, SH2B3*) that had previously been confirmed, and some rarer variants that could only be discovered once an adequately powered patient cohort had been studied (Fig. 1). Most of the genetic variants found had similar effects in both GD and Hashimoto thyroiditis (eg. *CTLA4, PTPN22, RNASET2*), but a small number including the *FLT3* variant appeared to predispose to Hashimoto thyroiditis but confer modest protection against GD. Although this study is the largest genetic study of AITD to date, it has to be considered that there were only 2,400 patients with GD analysed, compared to more than 27,000 with hypothyroidism or Hashimoto thyroiditis meaning there is a compromise in design, sacrificing the high power gained by having data from 30,000 individuals for the potential to miss important loci, as GD becomes a subphenotype of the analysis. Overall, it suggests that there may still be much to learn concerning the specific susceptibility to GD.

## Can genetic information inform clinical management in Graves’ disease?

While we have reviewed the genetic predisposition to GD above, important questions remain about whether there are the genotype-phenotype correlations, and if these genetic variations might have prognostic significance for patients. The phenotypic spectrum of GD is broad with patients exhibiting diverse clinical features and differing in biochemical severity. Several studies have demonstrated robust associations between specific clinical and biochemical factors and outcome in GD following withdrawal of antithyroid drugs (ATD), and have also identified prognostically relevant genetic associations.

Examining the association of genetic loci and clinical phenotype may not only provide mechanistic insight into the pathogenesis of GD, but also promote precision medicine by enabling the prediction of an individual’s risk of relapse and their likely response to different therapeutic approaches.

## Genotype-phenotype correlation in Graves’ disease

Candidate-gene association studies have revealed that some of the genetic variants associated with the predisposition to GD have also been associated with specific clinical and biochemical phenotypes. Several of these variants have also been implicated in predicting the outcome of GD, including both thyroid-specific (*TSHR* and *TG*) and immune-system genes (e.g. *CTLA4*, *CD40*, *PTNP22* and *HLA*) (Table 1).

**Table 1:** Studies demonstrating correlation between genotype and phenotype or treatment outcome of Graves’ disease

**Candidate gene**		**Potential mechanism of relapse**	**Genetic variant associated with outcome** **(risk allele, genotype)**	**Clinical phenotype(s) and/or associated with relapse**	**Immuno/biochemical phenotype(s) associated with relapse**	**Study population(s)**
Immune-modifying	*CTLA-4*	Decreased expression promoting T cell activation	rs231775 (GG)	Large goitre at end of treatment Smoking (Wang [69])	+ve TRAb at end of treatment	Taiwan (Wang et al. [59, 69])
			rs231775 (G, GG)	Not studied	+ve TRAb after 5 years of ATD treatment	Japan (Kinjo et al. [71])
			rs231775 (GG)	Younger at diagnosis (<30 years) Family history of GD GO (2^nd^ degree) Large goitre	Higher TRAb levels Lower Treg cells	Indonesia (Elian et al. [60])
			rs231775 (G, GG)	Younger age	None	Turkey (Tanrikulu et al. [61])
			rs231775 (GG)	Younger age	Lower TSH at end of treatment	Turkey (Sahin et al. [62])
			rs231775 (GG)	Presence of GO at diagnosis	None	Spain (Garcia-Mayor et al. [66])
			(G)	Increased susceptibility to thyroid associated orbitopathy	Not studied	UK Wellcome Trust (Vaidya et al. [28])
			(CC), (GG)	No significant association with relapse	No significant association with relapse	Korea (Kim et al. [72])
	*CD40*	Increased expression enhancing humoral immune activity	rs745307 (CT) rs11569309 (CT) rs3765457 (AG)	Large goitre at end of treatment Smoking	+ve TRAb at end of treatment	Taiwan (Wang et al. [69])
			(CC)	No significant association with relapse	No significant association with relapse	Korea (Kim et al. [72])
	*PTPN22*	Decreased expression promoting T cell activation	rs2476601 (CT)	Younger age (< 40 years) Enlarged goitre at diagnosis	Higher serum T4 at diagnosis (≥40 pmol/L) Higher TRAb at diagnosis (≥20 IU/L)	The Netherlands (Vos et al.[63])
			(T)	Younger age	No significant correlations	Poland (Skorka 2005)
	*HLA*	Enhanced autoantigen binding and presentation	DQB1*02 DQA1*05 DRB1*03	Younger age (<40 years) Enlarged goitre at diagnosis	Higher serum T4 at diagnosis (≥40 pmol/L) Higher TRab at diagnosis ( ≥20 IU/L)	The Netherlands (Vos et al. [63])
			DQA1*05	Longer course ATD	Higher serum T3 at diagnosis (21.5 ± 15.37 pmol/l)	Czech Republic (Vejrazkova et al. [70])
			rs2281388 (DPB1*0501), rs4947296, rs6903608 and rs6457617	Not studied	Persistent TRAb positivity	China (Chu et al. [49])
Thyroid-specific	*TG*	Dysregulation of thyroid hormonogenesis	E33SNP (CC)	Smoking Higher incidence of GO	+ve TRAb at end of treatment	Taiwan (Hsiao et al. [67])
			rs2069550 E10SNP158 (CC)	Not studied	Unable to replicate persistent +ve TRAb	Shangai (Gu et al. [51])
	*TSHR*	Negative effect on receptor stability inducing shedding of antigenic ectodomain	rs2268458 (CC)	Younger at diagnosis (<30 years) Family history of GD GO (2^nd^ degree) Large goitre	Higher TRAb levels Lower Treg cells	Indonesia (Eliana et al. [60])
			rs2239610 (CC)	Not studied	Higher serum FT4 and TRAb levels	Shangai (Gu et al. [51])
			rs12101261	Not studied	Persistent TRAb positivity	China (Chu et al. [49])

### Genetic variants and clinical/biochemical phenotype

Polymorphisms in both the immune-system and thyroid-specific genes have been associated with particular clinical and biochemical phenotypes. Some of the genetic variants independently associated with recurrence of GD are also associated with an earlier age of onset of GD (*CTLA4*, *HLA*, *PTPN22*, and *TSHR)* [60–63]. There are also limited data that implicate other genetic variants, such as an intronic *HCP5* polymorphism (rs3094228*)*, in younger onset GD. This polymorphism has been demonstrated to be associated in a dose-dependent manner, where the greater the number of *HCP5* risk alleles, the earlier the onset of GD [64, 65]. Despite these findings, the mechanistic link between these genes and younger onset GD remains to be established.

Other clinical features have been associated with genotype including an increased risk for, and severity of GO (*CTLA4*, *TSHR* and *TG)* [60, 66, 67] and a larger goitre size (*CTLA4*, *CD40*, *HLA*, *PTPN22*, and *TSHR)* [60, 63, 68, 69]. Polymorphisms in these genes have also been associated with a more severe biochemical phenotype, reflected by higher serum triiodothyronine (21.5 ± 15.37 pmol/l) (HLA DQA1*05)[70], thyroxine (≥40 pmol/L) (*PTPN22*)[63] and/or thyroid stimulating hormone antibody (TRAb) levels (*CTLA4*, CD40, *HLA*, *PTPN22*, *TG*, and *TSHR)* [60, 67–69]. The risk allele (G) and genotype (GG) of the *CTLA4* polymorphism, rs231775, have also been associated with the presence of persistently elevated TRAb levels after 5 years of ATD [71], suggesting that these patients may require a longer course of ATD or an alternative therapeutic approach.

## Genetic variants and outcome in Graves’ disease

Several of the genetic variants associated with particular clinical/biochemical phenotypes have also been independently associated with an increased risk of GD reoccurrence, albeit inconsistently across different study populations [63, 72, 73]. A Taiwanese study including 262 GD patients demonstrated that the risk allele (G) and genotype (GG) of the *CTLA4* polymorphism, rs231775, was associated with an increased risk of GD relapse 3 years after ATD withdrawal (relapse cases: 67% (GG) vs. 28% (AG) vs. 5% (AA)), with an odds ratio of 1.96 for the GG genotype compared with the combined group of GA plus AA genotypes [69]. This study also demonstrated association at three *CD40* polymorphisms (rs745307, rs11569309, rs3765457) with relapse of GD, with odds ratios of 7.9, 8 and 2.6 for risk vs. protective genotype, respectively [69].

Both *CTLA4* and *PTPN22* have key roles in regulating the T cell response, with both acting as negative regulators resulting in the inhibition of T cell activation [26, 29, 74–76]. Therefore, polymorphisms that decrease expression of *CTLA4* and *PTPN22* could potentially result in T cell proliferation and subsequent relapse. Indeed, the specific *CTLA4* (rs231775) and *PTPN22 (*rs2476601*)* polymorphisms associated with GD relapse, have been associated with increased T cell activation and proliferation [74], and a decreased frequency of the tolerogenic T regulatory cells [60, 77].

Conversely, CD40 which is a key co-stimulatory molecule expressed on antigen-presenting cells, drives T cell-dependent B cell activation [78]. Therefore, polymorphisms that may result in the increased expression of CD40 could potentially drive GD relapse through enhancing the humoral response. Indeed, haplotypes that include the *CD40* polymorphisms associated with outcome in GD have been associated with CD40 mRNA expression levels [79]. A precariously small study of 13 GD patients suggested that response to the novel therapeutic anti-CD40 monoclonal antibody, Iscalimab, might be predicted by haplotype [79]. Additionally, certain *HLA* haplotypes conferring susceptibility to GD and associated with disease outcome have been the target for novel therapeutics [63, 70, 80].

Variants in two thyroid specific genes, *TSHR* and *TG,* have been associated with outcome in GD. An Indonesian study of 144 GD patients demonstrated that those with the CC genotype of the intronic *TSHR* polymorphism, rs2268458, had a 13.3 times higher risk of relapse than those with the TT genotype [60]. The *TG* gene encodes the protein thyroglobulin which has a key role in thyroid hormone production and serum levels are often elevated in GD, where they have been associated with an increased risk of relapse [81]. The genetic variants in thyroid-specific genes may interact with the non-specific immune-related genes to work synergistically and influence the outcome in GD [82].

Identifying genetic variants that are associated with both clinical phenotype and prognosis may be clinically valuable to recognise patients at higher risk of relapse, and may help elucidate mechanistic insight into the functional impacts of genetic variation in GD.

## Predictive model

**Fig. 2:**
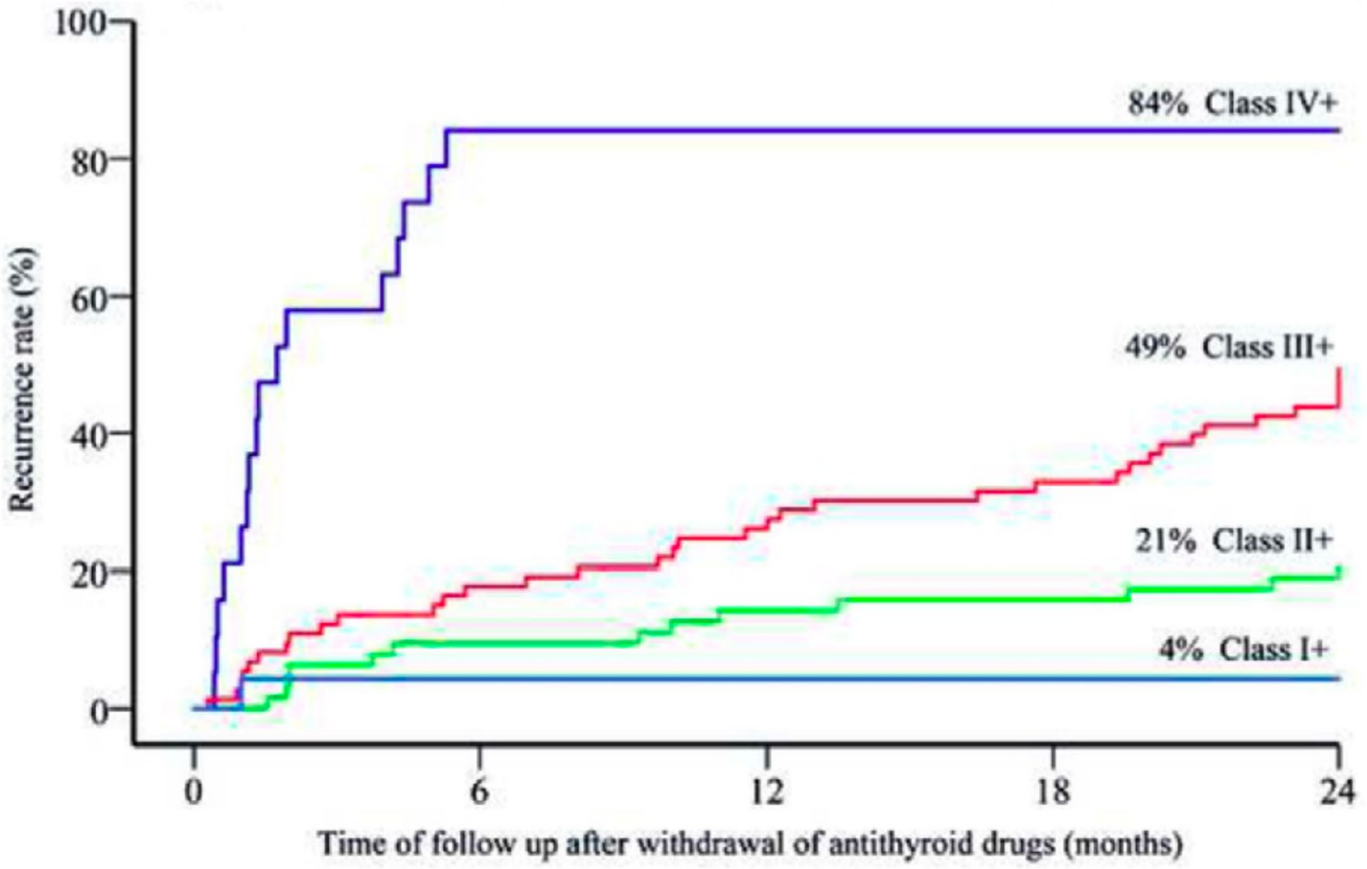
The GREAT+ score divides patients with Graves’ disease according to age, presenting serum free thyroxine concentration, TRAb antibody titre, and genotype for HLA class II variants, CTLA4 ‘CT60’ (rs3087243) and PTPN22 R620W (rs2476601) alleles (from Vos et al. reference 64)

A scoring model developed for predicting relapse of GD, ‘GREAT’ (Graves’ Recurrent Events After Therapy), uses specific clinical parameters (age, serum TRAb, goitre size) to stratify GD patients into different classes of recurrence risk. Despite the inconsistencies with genotype-phenotype correlations, the addition of genetic risk alleles at *HLA (DRB1-03, DQA1-05, DQB1-02)* and *PTPN22 (rs2476601)* to form the ‘GREAT+’ score, demonstrated an improved predictive value for relapse when compared to clinical factors alone (Fig. 2) [63]. The greatest benefit of using the GREAT+ score was described in those with a moderate risk of GD relapse (GREAT score class II), in whom the addition of genotyping significantly changed the recurrence risk (a change of approximately 25% was considered clinically relevant) and hence potentially the therapeutic approach used in 37 of 98 patients (38%) [63].

## Antithyroid drug and agranulocytosis

Antithyroid drugs are currently the mainstay of treatment in GD, but the rare side effect of agranulocytosis can be fatal. Studies have identified HLA-B*38:02 and HLA-DRB1*08:03 alleles as independent susceptibility loci for agranulocytosis with antithyroid drugs [83]. This may have clinical implications when deciding the best treatment approach for individuals at greater risk.

## Summary

Genotyping of GD patients may have significant translational potential by enabling a personalised treatment plan and the future implementation of novel immunomodulatory therapies for appropriate patients. However, the functional effects of these genetic variants in influencing prognosis remain largely unknown and the inconsistencies with genotype-phenotype correlations require larger GWAS studies to validate the candidate gene-association findings.

## Overall conclusion

Numerous genetic variants that predispose to Graves’ disease have now been identified in robust studies in the Chinese population, as well as in smaller cohorts of people of white European ancestry. Inheritance appears to be truly polygenic with upwards of 80 loci identified, most of which contribute a small increased risk of disease typically around a 10-20% change in risk. Several studies have already correlated clinical features such as relapse following ATD cessation or age of onset of hyperthyroidism with specific genetic variants but much larger cohorts of patients followed for longer periods of time are now needed to determine more detailed phenotypic associations with known and unknown genetic variants that could prove clinically helpful for a precision medicine approach to GD management.

## Data Availability

No original or unpublished data are contained in this paper.

## Abbreviations

*ADCY7*: Adenylyl Cyclase Type 7
AITD: Autoimmune Thyroid Disease
*ATD*: Anti-Thyroid Drugs
*BACH2*: Broad Complex-Tramtrack-Bric a Brac and Cap'n'collar Homology 2
*C1QTNF6*: Complement C1q Tumour Necrosis Factor-Related Protein 6
*CD40*: Cluster of Differentiation 40
Csk: C-terminal Src kinase
CTLA4: Cytotoxic T-lymphocyte Antigen-4
*FCRL3*: Fc Receptor-Like Protein 3
*FLT3*: Fms Related Receptor Tyrosine Kinase 3
FT3: Free Trioiodothyronine
FT4: Free Thyroxine
GD: Graves’ disease
GM-CSF: Granulocyte-Macrophage Colony-Stimulating Factor
GO: Graves' Ophthalmopathy
*GPR174*: G Protein-Coupled Receptor 174
GREAT: Graves’ Recurrent Events After Therapy
GWAS: Genome-Wide Association Study
*HCP5*: HLA Complex P5
HLA: Human Leukocyte Antigen
IL: Interleukin
LYP: Lymphoid Tyrosine Phosphatase
MHC: Major Histocompatibility Complex
MHC-DRβ: Major Histocompatibility Complex - DRβ isotype
mRNA: Messenger Ribonucleic Acid
PTPN22: Protein Tyrosine Phosphatase Non-receptor type 22
RAC2: Ras-related C3 botulinum toxin substrate 2
*RNASET2*: Ribonuclease T2
SCGB3A2: Secretoglobin Family 3A Member 2
*SH2B3*: SH2B adaptor protein 3
SNP: Single-Nucleotide Polymorphism
T3: Triiodothyronine
T4: Thyroxine
TCR: T cell Receptor
TED: Thyroid eye disease
TG: Thyroglobulin
Treg: Regulatory T cells
TSH: Thyroid Stimulating Hormone
TSHR: Thyroid Stimulating Hormone Receptor
UGRP1: Uterus Globulin Associated Protein-1
UK: United Kingdom
US: United States
WTCCC: Wellcome Trust Case Control Consortium
λs: sibling recurrence risk ratio
+ve: positive
-ve: negative
